# Dual-Polarizer Linearly Polarized Light Alters Wavelength- and *E*-Vector-Dependent Aggregation Responses in *Locusta migratoria manilensis*

**DOI:** 10.3390/insects17080859

**Published:** 2026-08-18

**Authors:** Qihang Liu, Likui Wang, Fu Wang, Jinhai Yang, Fen Li, Min Li

**Affiliations:** 1 Henan Institute of Science and Technology, Xinxiang 453003, China; bjliuqihang@163.com; 2 School of Breeding and Multiplication, Sanya Institute of Breeding and Multiplication, Hainan University, Sanya 572025, China; 19937157228@163.com (L.W.); wf18534706883@163.com (F.W.); yjinhai0513@163.com (J.Y.); lifen2010happy@sina.com (F.L.); 3 Technology Center of Kunming Customs, Kunming 650228, China

**Keywords:** *Locusta migratoria manilensis*, linearly polarized light, polarization angle, polarotactic aggregation, behavioral assay, rearing shed

## Abstract

Light-based trapping is a promising non-chemical approach for sustainable locust management in protected agriculture. However, how polarized light properties influence locust aggregation behavior under shed conditions remains insufficiently characterized. To clarify how polarized spectral properties and polarized light configurations, namely linearly polarized light (LPL) and linear detection-polarized light (LDPL), modulate polarotactic aggregation sensitivity, and to support the development of polarized spectral trapping devices for locust control, this study systematically evaluated the responses of *Locusta migratoria manilensis* to violet light (405 nm) and orange light (610 nm) based on prior laboratory findings. Locusts were exposed to LPL and LDPL with different *E*-vector orientations (0–330° at 30° intervals) under different nocturnal illumination durations. The results showed that differences in aggregation sensitivity between the two polarized light configurations were wavelength-dependent. Under violet light, the largest sensitivity differences occurred at the 150° *E*-vector orientation for LPL and the 240° *E*-vector orientation for LDPL, whereas under orange light, the largest differences were observed at the 270° *E*-vector orientation for LPL and the 240° *E*-vector orientation for LDPL. The duration-dependent enhancement of aggregation sensitivity also exhibited wavelength-dependent temporal threshold characteristics. After 10 h of illumination, LPL induced the highest mean aggregation sensitivity at the 270° *E*-vector orientation under both violet and orange light, whereas LDPL produced the highest mean aggregation sensitivity at the 240° *E*-vector orientation under violet light and at the 30° *E*-vector orientation under orange light. The spectral properties of LPL did not alter the cosine-periodic response of locusts to the *E*-vector, with sensitivity-associated *E*-vector orientations occurring at 270°, 180°, 0°, and 90°. In contrast, the spectral properties of LDPL reshaped the wavelength-dependent sine- and cosine-like tuning patterns and markedly shifted the sensitivity-associated *E*-vector orientations, with prominent orientations of 240° and 270° under violet light and 30° and 240° under orange light. Notably, under violet light, LDPL elicited significantly stronger light-induced aggregation sensitivity than LPL, with the highest observed response occurring at the 240° *E*-vector orientation after 10 h of illumination. Moreover, the polarized light configuration modulated the combined effects of polarized spectral properties and illumination duration: the duration-dependent enhancement was more pronounced under orange light, whereas the overall light-induced aggregation response was stronger under violet light. Collectively, these findings demonstrate that polarized spectral properties and polarized light configurations jointly determine locust polarotactic aggregation sensitivity, with linear detection polarized violet light producing the highest induction effect under specific *E*-vector and temporal conditions. This study reveals the sine- and cosine-like tuning characteristics of locust responses to different polarized light configurations, together with their wavelength dependence and temporal threshold effects, thereby providing a theoretical basis and behavioral evidence for the development of efficient polarized spectral trapping devices.

## Simple Summary

Locust outbreaks can cause serious crop losses, and farmers need trapping methods that reduce reliance on chemical pesticides. Many insects can see polarized light, which is light whose waves are aligned in a particular direction, but it is unclear how this type of light can be used to guide locust behavior under rearing-shed conditions. Based on previous findings, this study tested adult Oriental migratory locusts exposed at night to violet or orange polarized light produced with either one polarizing filter or two filters. The locusts gathered differently depending on light color, polarization direction, light source configuration, and exposure time. Violet light generally attracted stronger aggregation than orange light. With one filter, the strongest responses occurred when the polarization direction was 270°, whereas with two filters the strongest responses occurred at 240° under violet light and 30° under orange light. Aggregation responses increased progressively with illumination duration and peaked at 05:30 (after 10 h). These findings show that carefully selected polarized light settings can strengthen locust aggregation and may help design more efficient light-based traps for sustainable locust management.

## 1. Introduction

Against the backdrop of global climate change and ecological deterioration, agricultural pest outbreaks are becoming increasingly frequent, sudden, and difficult to predict. Locusts are among the most destructive agricultural pests worldwide, posing serious threats to crop and livestock production, the environment, and regional economies. Although current locust management strategies still rely largely on chemical pesticides, their intensive and repeated use has raised concerns about agricultural production safety and environmental risks [1,2]. Polarized light-based trapping may offer an environmentally friendly physical control strategy for locust management. This strategy is based on locust polarization perception and polarization-sensitive vision behavior, as well as factors that influence phototactic and aggregation responses [3,4]. By identifying polarized light types that effectively induce polarotactic aggregation and by defining the technical characteristics of polarized spectral light fields, this approach aims to support polarized light-mediated trapping and control of locusts. Such research is closely related to the development and application of new equipment for protected agriculture, it is consistent with the concept of green pest management and has practical significance for food security and human health.

In insect vision research, polarization sensitivity refers to the ability of insects to use polarized light for navigation and orientation [5]. Previous studies have shown that interactions among different spectral classes of photoreceptors in the locust compound eye contribute to polarization sensitivity [6,7,8]. The dorsal rim area (DRA) exhibits substantially higher polarization sensitivity than non-DRA regions, but non-DRA regions can also respond to polarization vector information. Together, the polarization-sensitive arrays distributed across the retina form an integrated polarization-sensitive visual system that supports polarization-based orientation, navigation, and related biological behaviors. Further studies have shown that locust responses to unpolarized light depend on both the stimulated region of the compound eye and the wavelength of the light stimulus, whereas responses to polarized light depend on the spectral tuning properties of ultraviolet-, blue-, and green-sensitive photoreceptors in the DRA [9,10]. The locust central complex integrates polarized light input from the DRA with color and intensity information from non-DRA regions, contributing to polarization-based orientation behavior. Therefore, spatial orientation and navigation in locusts require the perception and integration of multiple environmental cues. From the perspective of light field properties, locust polarization perception is closely associated with spectral illumination characteristics, whereas the polarization vector pattern plays a decisive role in polarotactic orientation [11,12]. This indicates that spectral light and polarized light have distinct but interacting effects on polarization-sensitive vision in locusts. Moreover, the combined effects of wavelength perception, light intensity perception, and polarization vision can alter locust directional sensitivity, preference selection, and taxis responses to polarized illumination. Consequently, the effects of polarized spectra and polarization characteristics, including intensity and *E*-vector direction, on polarization vector-sensitive response patterns in locusts are strongly dependent on the visual environment [13,14]. In natural habitats, locusts are often exposed to complex polarized light fields generated by multiple reflection and scattering processes, such as light reflected from water surfaces or scattered by vegetation [15]. These light fields differ substantially from linearly polarized light with a single *E*-vector direction. In this study, LPL (linearly polarized light) refers to light transmitted through a single linear polarizer, whereas LDPL (linear detection-polarized light, dual polarizer-modulated linearly polarized light) refers to a dual polarizer configuration consisting of a fixed 0° polarizer followed by a second analyzer set to a defined transmission axis angle. LDPL is therefore an optical path configuration rather than a distinct polarization state. Although the output of LDPL remains linearly polarized, both its *E*-vector direction and light intensity can be modulated by the second polarizer, allowing this configuration to approximate heterogeneous polarized light environments.

It should be emphasized that LPL and LDPL do not represent two fundamentally different polarization states; rather, they denote two different optical path configurations or polarized light configurations. Clarifying the behavioral differences between these configurations is important for identifying polarized light parameters that may enhance locust aggregation. However, existing studies on locust polarotactic sensitivity have mainly been conducted under a single polarized light condition in laboratory settings [16]. The differential sensitivity and response characteristics of locusts to different polarized light configurations in field shed environment remain poorly documented. This knowledge gap hinders the identification of preferred polarized light configurations and optimal *E*-vector orientations for inducing locust polarotactic aggregation, thereby limiting the practical application of polarized light induction technologies for locust control.

From the perspective of the coding properties of the locust polarization vision system [17], LPL and LDPL can be expected to induce different behavioral responses. Although both stimuli are physically linearly polarized, they differ in two key optical features relevant to visual encoding. First, LPL provides a direct *E*-vector cue determined by the transmission axis of a single polarizer, whereas LDPL introduces an analyzer-dependent configuration in which the emergent *E*-vector is determined by the second polarizer. Second, LDPL couples changes in analyzer orientation with light intensity attenuation according to Malus’ law [18], whereas LPL does not produce analyzer angle-dependent intensity modulation. Previous studies have shown that the dorsal rim area (DRA) of the locust compound eye is finely tuned to polarization direction, whereas non-DRA regions are sensitive to changes in light intensity [19]. Neurons in the locust central complex can integrate polarization direction and light intensity information to generate orientation-related behavioral outputs. Thus, even when the two stimuli share the same polarization type, differences in *E*-vector presentation and light intensity dynamics may be encoded by the locust visual system as different stimulus information, thereby inducing differential polarotactic aggregation responses. In addition, locust polarization-vision responses are time dependent. The cumulative effect of illumination duration may regulate the sensitivity threshold of the locust visual system and influence the encoding and integration efficiency of polarized light signals [20]. Therefore, nocturnal illumination duration is an important variable regulating locust polarotactic aggregation sensitivity, and locust responses to different polarized light configurations may exhibit different optimal time windows.

Based on these considerations, we hypothesized that locust aggregation responses differ between LPL and LDPL configurations and that these differences depend on wavelength, *E*-vector orientation and nocturnal exposure duration. In LDPL, light intensity varies with analyzer orientation according to Malus’ law, thereby generating a fixed functional coupling between *E*-vector orientation and light intensity. In contrast, LPL maintains relatively constant light intensity across different *E*-vector orientations. The direction–intensity coupling in LDPL and the direction–intensity constancy in LPL therefore define two different stimulus regimes. Because non-DRA regions of the locust compound eye are sensitive to light intensity, the DRA is tuned to polarization direction, and the central complex integrates these two streams of visual information [21], the coupled optical features of LDPL may be encoded by the locust visual system as signals resembling those generated by heterogeneous polarized light environments. This encoding process may, in turn, alter the periodic response characteristics of locusts to *E*-vector orientation.

To test this hypothesis, violet and orange LPL and LDPL sources were developed, and the polarotactic aggregation sensitivity of *Locusta migratoria manilensis* under vector-controlled polarized illumination was examined under shed conditions. Specifically, differences in aggregation sensitivity and response dynamics between the two optical path configurations were compared to identify the preferred polarized light configuration and optimal *E*-vector orientation for inducing locust aggregation in field-like habitats. The response patterns and behavioral characteristics of locust polarotactic aggregation were further characterized, and the key factors governing polarized light-mediated aggregation, together with their potential mechanisms of action, were explored. These findings are expected to provide a theoretical and technical basis for developing intelligent polarized spectral light sources for locust control, as well as a reference for elucidating the mechanisms underlying insect orientation responses to heterogeneous polarized light configurations.

## 2. Materials and Methods

### 2.1. Rearing Conditions and Experimental Location

The experiment assessing the polarotactic aggregation response of locusts to linearly polarized light was conducted from July to August 2024 in a gauze-covered rearing shed at the Fanxiang Locust Breeding Base, Fanxian County, Puyang, Henan Province, China (35.80° N, 115.49° E). The shed measured 30 m × 8 m × 4 m (length × width × height).

The test locusts, *L. migratoria manilensis*, were reared for multiple generations within the gauze-covered shed. The shed structure consisted of a steel frame covered with 40-mesh white nylon gauze, which ensured natural ventilation and lighting while preventing insect escape and ingress of natural predators. Wheat and corn seedlings were planted on the ground inside the shed as a natural food source and were reseeded periodically. Fresh corn leaves and bran were supplemented daily as additional feed. A spray system was used to provide water twice daily, in the morning and evening. The rearing density was maintained at approximately 200–300 individuals per square meter. During the experimental period (July to August), the daytime temperature inside the shed ranged from 28 °C to 38 °C, and the nighttime temperature ranged from 22 °C to 28 °C; the relative humidity was maintained between 45% and 70%. Prior to the experiments, healthy adults with intact appendages and normal activity were collected 5–7 days after emergence for behavioral testing, with a male-to-female ratio of approximately 1:1.

The insects used were healthy adults of *L. migratoria manilensis* that had been artificially reared for multiple generations within the breeding shed and had emerged within 5–7 days prior to the experiments. Their body size and developmental stage were visually confirmed to be largely uniform across the experimental population. The average density of locusts in the experimental shed was estimated as 270 ± 15 individuals m^−2^ (mean ± SD), calculated by counting the number of locusts within 10 randomly selected 1 m × 1 m quadrats before the start of each experimental cycle, with the mean value averaged across all six cycles (*n* = 10 quadrats × 6 cycles).

### 2.2. Experimental Light Sources and Shed Layout

**Figure 1:**
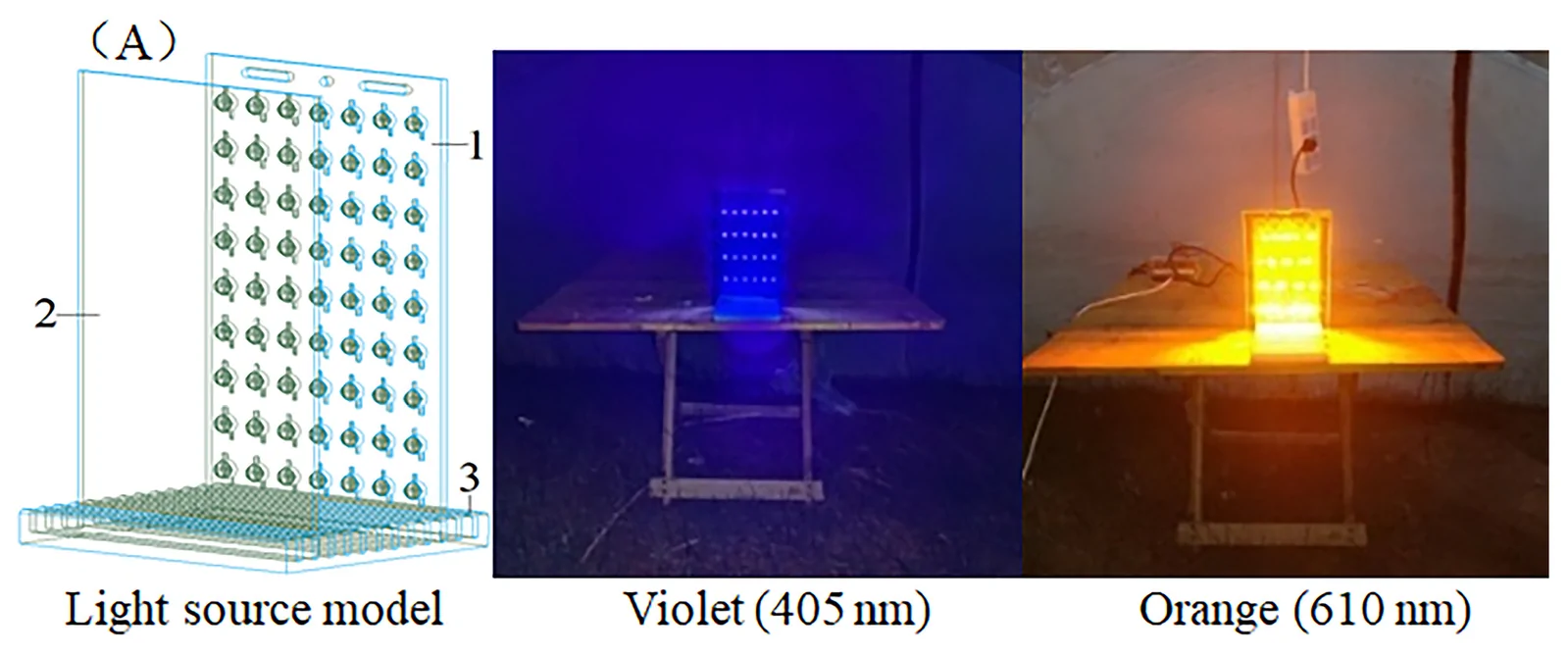

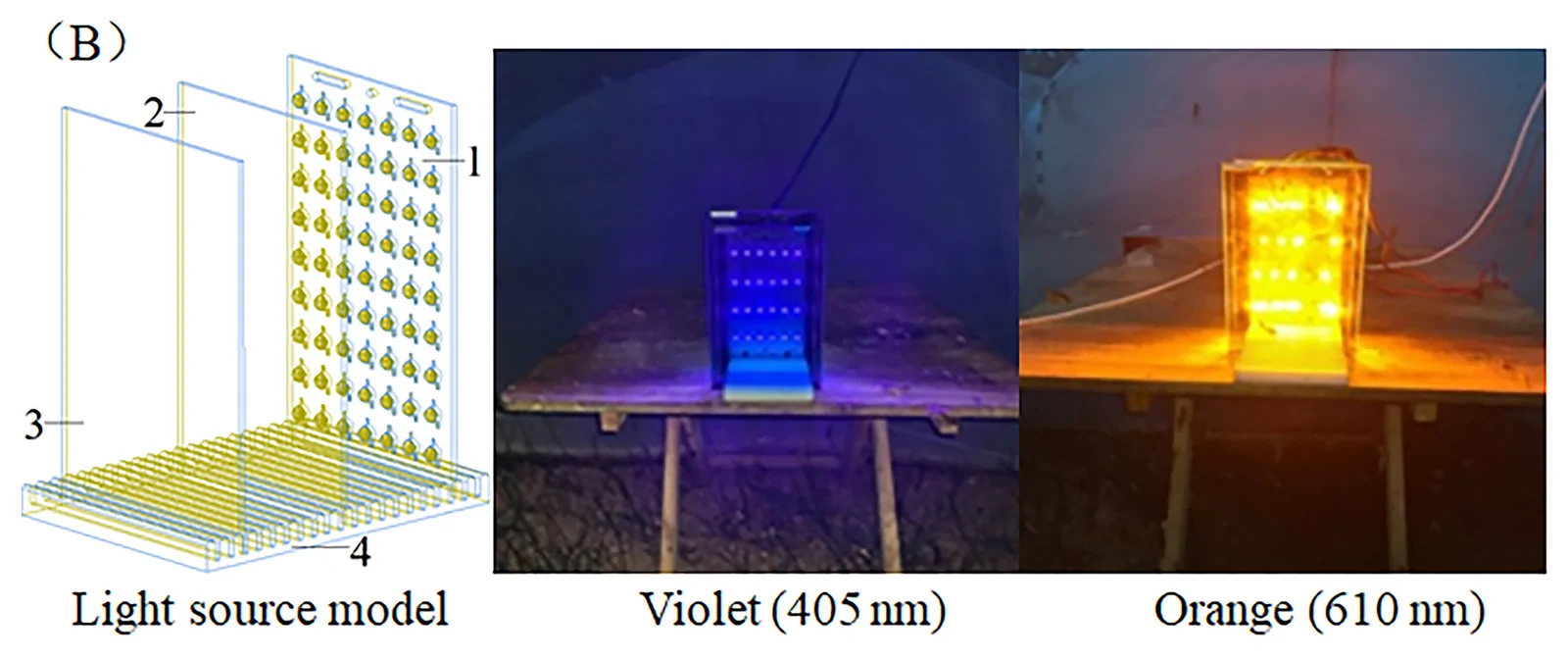
Experimental light sources and emission spectra. (**A**) Linearly polarized light source and the spectra used for testing: 1. spectral light source; 2. linear polarizer; 3. support. (**B**) linear detection-polarized light source and the spectra used for testing: 1. spectral light source; 2. linear polarizer with 0° vector; 3. polarized detection polarizer with different vectors; 4. support.

To evaluate the polarotactic aggregation sensitivity of locusts to LPL and LDPL, polarized spectral light sources capable of producing both LPL and LDPL were developed (Figure 1A,B). Two spectra were used in the experiment: violet light with a peak wavelength of 405 nm and orange light with a peak wavelength of 610 nm. The selection of violet (405 nm) and orange (610 nm) light was based on previous laboratory studies examining locust polarotactic responses to monochromatic polarized light across the visible spectrum, including violet, blue, green, and orange wavelengths [22]. These studies demonstrated that violet light induced the strongest phototactic aggregation sensitivity, whereas orange light was most effective in modulating polarization vision sensitivity. Preliminary trials conducted in the rearing-shed environment further confirmed that other spectral bands produced considerably weaker induction effects. The two wavelengths are widely separated in the spectrum, providing an ideal contrast for investigating spectral–polarization coupling and effectively avoiding confounding effects that could arise from wavelength proximity and LPL and LDPL served as mutual controls under the same spectral condition to evaluate the regulatory differences between the two polarized light configurations. A dark control, in which no experimental light source was turned on, and only nocturnal natural background light (<0.1 l×) was retained, was conducted to exclude the influence of endogenous circadian rhythm on locust activity.

Three-watt light-emitting diodes (LEDs; Hongtai Electronics Co., Ltd., Shenzhen, China) were soldered onto 2 mm thick aluminum substrates (200 mm × 120 mm, length × width) to fabricate twelve 9 × 7 spectral LED arrays, comprising six violet and six orange light sources. As shown in Figure 1, each spectral light source was mounted on a support frame and driven by a 12 V DC power supply. At an operating voltage of 12 V, the illuminance of the violet and orange light sources was adjusted to 30,000 l× and 300,000 l×, respectively, using an illuminance meter (XRP-3000; resolution, 0.01 l×; Shenzhen Ouya Precision Instruments Co., Ltd., Shenzhen, China).

In the LPL configuration (Figure 1A), a 3 mm thick linear polarizer (PL-CIR, HOYA, Tokyo, Japan; 210 mm × 130 mm, length × width; transmittance, 50%; polarization efficiency, 95%; extinction ratio, 1000:1) was placed in the support frame slot 10 cm in front of the spectral light source to generate linearly polarized light. Twelve linear polarizers with transmission axis orientations of 0° (360°), 30°, 60°, 90°, 120°, 150°, 180°, 210°, 240°, 270°, 300° and 330° were used to produce linearly polarized illumination with different *E*-vector orientations for testing.

In the LDPL configuration (Figure 1B), a 3 mm thick linear polarizer with a 0° transmission axis orientation (PL-CIR, HOYA, Japan; 210 mm × 130 mm, length × width; transmittance, 50%; polarization efficiency, 95%; extinction ratio, 1000:1) was first fixed 10 cm in front of the spectral light source to generate a linearly polarized beam. A 3 mm thick linear analyzer (PL-CIR, HOYA, Japan; 220 mm × 140 mm, length × width; transmittance, 50%; polarization efficiency, 95%; extinction ratio, 1000:1) was then mounted in the support frame slot 10 cm in front of the 0° polarizer. This optical assembly, consisting of the spectral light source, the 0° linear polarizer, and a vector-controlled linear analyzer, generated the LDPL used in the experiment. Twelve analyzers with transmission axis orientations of 0° (360°), 30°, 60°, 90°, 120°, 150°, 180°, 210°, 240°, 270°, 300° and 330° were used to produce linear detection-polarized light with different *E*-vector orientations for testing.

**Figure 2:**
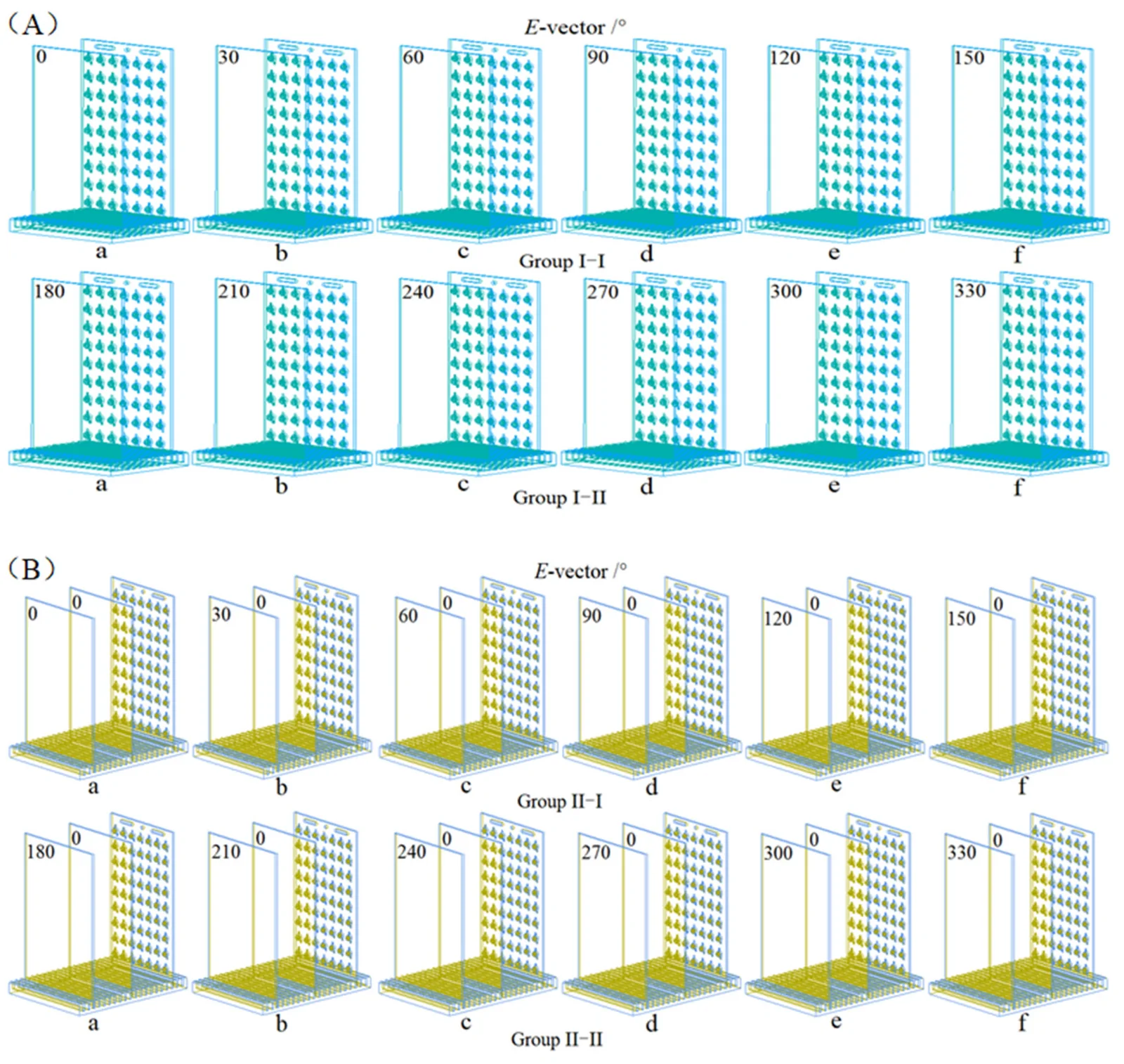
Arrangement of the experimental light sources in the locust rearing shed. (**A**) Arrangement of the linearly polarized light source; (**B**) arrangement of the linear detection-polarized light source. (**A**) Groups I-I and I-II represent the first and second groups of linearly polarized light sources, respectively; (**B**) Groups II-I and II-II represent the first and second groups of linear detection-polarized light sources, respectively. In both panels, labels (**a**–**f**) denote six light sources with different *E*-vector orientations within each group: (**a**) = 0° (360°), (**b**) = 30°, (**c**) = 60°, (**d**) = 90°, (**e**) = 120°, and (**f**) = 150° for Group I-I and Group II-I; for Group I-II and Group II-II, (**a**) = 180°, (**b**) = 210°, (**c**) = 240°, (**d**) = 270°, (**e**) = 300°, and (**f**) = 330°. The six rearing sheds are labeled I–VI, and the light sources were sequentially rotated among sheds I–VI during the experiment to ensure six replicate trials for each *E*-vector condition.

For the six locust rearing sheds used in the experiment (labeled I–VI), six LPL sources with the same spectrum and *E*-vector orientations ranging from 0° to 150° (Figure 2A: Group I-I, labeled a–f) were installed at the front-center position of the sheds, with one source assigned to each shed for testing (Figure 2A). During the six-day experimental period, the six LPL sources were sequentially rotated among sheds I–VI, ensuring that each vector-controlled LPL source was tested once in each shed. The same procedure was subsequently applied to six LPL sources with *E*-vector orientations ranging from 180° to 330° (Figure 2A: Group I-II, labeled a–f), which were also rotated among sheds I–VI over six days; thus, each LPL *E*-vector condition was evaluated in six replicate trials. Using the same spatial arrangement and experimental duration, two sets of six LDPL sources with the same spectrum, with *E*-vector orientations ranging from 0° to 150° and from 180° to 330°, were assigned to Groups II-I and II-II (Figure 2B: labeled a–f), respectively. These LDPL sources were sequentially rotated among sheds I–VI following the same protocol, thereby ensuring six replicate trials for each vector-controlled LDPL condition.

During the experiment, environmental conditions inside the locust rearing sheds were monitored using a temperature and humidity recorder (WSZY-1; Beijing Tianjian Huayi Technology Co., Ltd., Beijing, China), and the actual temperature and relative humidity were continuously recorded throughout the experimental period. The shed temperature was maintained at 28 ± 2 °C, and the relative humidity was maintained at 60 ± 5%. To evaluate the polarotactic aggregation sensitivity of locusts under realistic nocturnal conditions, natural background light inside the sheds was not completely excluded during the experimental period from 19:30 to 05:30; instead, the experimental polarized light sources were introduced as supplementary light stimuli. Before each trial, the baseline illuminance inside the sheds was measured using an illuminance meter (XRP-3000; resolution, 0.01 l×; Shenzhen Ouya Precision Instruments Co., Ltd., Shenzhen, China). The nocturnal natural illuminance inside the sheds was below 0.1 l×, which was far lower than the illuminance of the experimental light sources, ensuring that the experimental light sources served as the dominant stimuli regulating nocturnal polarotactic behavior in locusts.

### 2.3. Experimental Methods

Based on the vegetation coverage and locust density distribution within the six rearing sheds, one light source was installed at the front end of each shed and positioned 500 mm above the ground to ensure an effective induction effect. Considering the behavioral characteristics of locust polarotactic aggregation and the configuration of the light source, a marker line was established 1 m in front of each light source. The number of locusts attracted to the light source was recorded within a 2 m^2^ observation area, defined as a 2 m wide zone extending 1 m to the left and 1 m to the right of the light source, and 1 m forward from the source. Before the experiment, six LPL sources with the same spectrum but different *E*-vector orientations in Group I-I were assigned to sheds I–VI. During each trial, the light sources were switched on at 19:30 and switched off at 05:30 the following morning. Locusts within the marked area were counted and photographed at 21:30, 23:30, 01:30, 03:30 and 05:30 each day. Each light source was sequentially rotated among sheds I–VI over a six-day experimental period, ensuring that each source was tested once in each shed and yielding six replicate trials per light source. The number of replicates was determined according to the experimental cycle, in which each light source was tested once in each of the six sheds during the six-day test period assigned to each light source group. A power analysis based on preliminary experimental results indicated that, at α = 0.05, power = 0.80 and an effect size of d = 0.6, a minimum of five replicates was required. Therefore, six replicates were used for each treatment in the present study, meeting the statistical requirement and reducing the influence of random experimental error. After completion of the Group I-I trials, six LPL sources with the same spectrum but different *E*-vector orientations in Group I-II were tested in sheds I–VI using the same procedure, and this experiment was completed within six days. Following the LPL trials for Groups I-I and I-II, six LDPL sources with the same spectrum but different *E*-vector orientations in Groups II-I and II-II were sequentially tested in sheds I–VI using the same experimental procedure. Under both violet and orange spectral conditions, the six light sources with different *E*-vector orientations in Groups I-I, I-II, II-I and II-II were sequentially tested in sheds I–VI following the same protocol.

To assess potential temporal changes in locust activity unrelated to the experimental polarized light stimuli, a dark-control treatment was conducted under the same observation schedule. In this treatment, no experimental polarized light source was turned on, and only the nocturnal natural background light inside the shed was retained. After shading cloth was applied, the illuminance inside the shed was below 0.1 l×, as measured using an XRP-3000 illuminance meter. The experimental duration, observation area (2 m × 1 m), and observation time points (21:30, 23:30, 01:30, 03:30 and 05:30) were identical to those used in the polarized light trials. At each time point, six replicate counts were performed. The number of active locusts within the marked area was recorded, and the activity density was calculated using the same method and expressed as individuals m^−2^. The dark-control results showed no significant temporal variation in locust activity density between 21:30 and 05:30 under non-illuminated conditions, indicating that endogenous circadian activity remained relatively stable during this period. Accordingly, the differences in polarotactic aggregation sensitivity observed across time points in the illumination trials were mainly attributable to the cumulative effect of illumination duration under the polarized light sources, rather than to interference from the endogenous circadian rhythm of locusts. Therefore, the influence of endogenous circadian rhythm was not further considered in the subsequent Section 3 and Section 4.

### 2.4. Data Processing and Statistical Analysis

To quantify the polarotactic aggregation response of locusts to different polarized light sources, locust polarotactic aggregation density (*ρ*) was defined as the evaluation index. This index reflects the net aggregation effect induced by the light source after excluding the influence of the initial locust distribution density. It was calculated as follows:

For each light source in each rearing shed, the number of locusts attracted to the marked area in front of the light source (2 m × 1 m = 2 m^2^) was counted across six replicate trials, and the mean value was calculated as N_mean_. The induced locust density per unit area was then obtained by dividing the mean number of attracted locusts by the observation area S, where S = 2 m^2^ in this study; thus,

D_induced_ = N_mean_/S = N_mean_/2, (1)

expressed as individuals m^−2^. To eliminate the influence of the initial locust distribution density in the shed on the estimated aggregation response, the initial density N_0_, measured before the experiment and set at 270 individuals m^−2^, was subtracted from D_induced_. The resulting value was defined as the polarotactic aggregation density ρ and was calculated as

*ρ* = D_induced_ − N_0_ = N_mean_/S − N_0_, (2)

Because S = 2 m^2^, the equation was simplified to

*ρ* = N_mean_/2 − 270, (3)

with the unit of individuals m^−2^.

In this equation, a higher *ρ* value indicates greater polarotactic aggregation sensitivity of locusts to the corresponding light source. Based on this method, the polarotactic aggregation density of locusts under LPL was denoted as *ρ*_1_, whereas that under LDPL was denoted as *ρ*_2_.

To compare the light-induced effects of LPL and LDPL, the difference in locust polarotactic aggregation density between the two polarized light configurations was calculated. Depending on the direction of comparison, this difference was expressed as either

Δ*ρ* = *ρ*_LDPL_ − *ρ*_LPL_, (4)

Positive Δ*ρ* values indicate stronger aggregation under LDPL, whereas negative values indicate stronger aggregation under LPL. Because locusts exhibited consistent polarotactic aggregation patterns in response to LPL and LDPL with the same *E*-vector orientation across nocturnal observation time points, the mean polarotactic aggregation density over the full nocturnal illumination period from 19:30 to 05:30 was used to characterize the response patterns and light-induced effects of vector-controlled LPL and LDPL illumination. Furthermore, based on the comparative results between LPL and LDPL and the observed polarotactic aggregation characteristics, *E*-vector orientations that induced relatively strong aggregation responses were selected for further analysis. Specifically, the aggregation densities induced by LPL and LDPL at *E*-vector orientations of 0°, 30°, 90°, 150°, 180°, 240° and 270° were used to evaluate the polarotactic aggregation sensitivity of locusts to vector-controlled polarized light illumination.

Prior to all parametric statistical analyses, the normality of data distributions was assessed using the Shapiro–Wilk test (*W* > 0.90, *p* > 0.05 for all datasets), and homogeneity of variances was verified using Levene’s test (*F* < 3.50, *p* > 0.05 for all comparisons). These assumptions were satisfied for all datasets analyzed in this study. All experimental data were processed and statistically analyzed using Excel and SPSS 16.0 (SPSS Inc., Chicago, IL, USA), with additional analyses performed using custom functions in MATLAB (Version 2021a; The MathWorks, Natick, MA, USA). Under the same spectral condition, one-way analysis of variance (one-way ANOVA) within the general linear model framework was used to evaluate differences in light-induced locust polarotactic aggregation among *E*-vector orientations at the same nocturnal observation time, among nocturnal observation times under the same *E*-vector orientation, and among the difference values in polarotactic aggregation density between LDPL and LPL treatments. Post hoc pairwise comparisons were conducted only when the overall ANOVA was statistically significant (*p* < 0.05), using Tukey’s HSD (Honestly Significant Difference) test for post hoc pairwise comparisons following significant ANOVA results, with the significance level set at α = 0.05. Student’s *t*-test was used to assess significant differences between spectral treatments or between polarized light configuration treatments under the same *E*-vector orientation and nocturnal observation time, also at *p* = 0.05. All results are presented as the mean ± standard error (SE).

## 3. Results

### 3.1. Differences in Locust Polarotactic Aggregation Sensitivity Between LPL and LDPL

Under identical nocturnal illumination durations, *E*-vector orientation significantly modulated the difference in locust polarotactic aggregation sensitivity between linearly polarized light (LPL) and linear detection-polarized light (LDPL), and the magnitude of this modulation varied with the spectral properties of the light source (Table 1 and Table 2). Under violet light (Table 1), the effect of *E*-vector orientation was strongest at 03:30 (*F* = 101.583, *p* < 0.001) and weakest at 01:30 (*F* = 30.134, *p* < 0.001). Pairwise comparisons between LDPL and LPL showed that LPL elicited stronger aggregation responses than LDPL at *E*-vector orientations of 30°, 60°, 120°, 150° and 330°; Among these orientations, the sensitivity difference was largest at 150° (*F* = 14.87, *p* = 0.000), where the LPL-dominant effect was statistically significant. At 330°, the numerical difference was smaller, but this difference was not statistically significant (*F* = 0.334, *p* = 0.849) (Table 1). At the other *E*-vector orientations, LDPL elicited stronger aggregation responses than LPL, with the largest sensitivity difference observed at 240° and the smallest at 180°. Under orange light (Table 2), the effect of *E*-vector orientation was most significant at 03:30 (*F* = 530.024, *p* < 0.001) and weakest at 21:30 (*F* = 43.996, *p* < 0.001). Comparisons between LPL and LDPL indicated that LDPL elicited stronger aggregation responses than LPL at *E*-vector orientations of 30°, 90° and 240°; among these orientations, the LDPL-dominant sensitivity difference reached statistical significance only at 240° (*F* = 3.493, *p* = 0.049), whereas the smaller numerical difference observed at 90° was not statistically significant (*F* = 0.657, *p* = 0.635) (Table 2). At the remaining *E*-vector orientations, LPL elicited stronger aggregation responses than LDPL, with the largest sensitivity difference observed at 270°. The smallest difference among the remaining LPL-dominant orientations should be interpreted according to the numerical ranking in Table 2.

The effect of nocturnal illumination duration on the difference between LPL- and LDPL-induced aggregation responses varied with both *E*-vector orientation and the spectral properties of the light source. Under violet light (Table 1), illumination duration had a significant effect only at the 150° *E*-vector orientation (*F* = 14.87, *p* < 0.001), whereas no significant effect was detected at the other *E*-vector orientations (*p* > 0.05). The weakest effect of illumination duration was observed at 300° (*F* = 0.062, *p* = 0.992), followed by 120° (*F* = 0.081, *p* = 0.984). Under orange light (Table 2), illumination duration significantly affected the difference between LPL- and LDPL-induced aggregation responses at *E*-vector orientations of 240° and 270° (*F* = 3.493, *p* = 0.049; *F* = 6.316, *p* = 0.008, respectively), whereas no significant effect was detected at the other *E*-vector orientations (*p* > 0.05). The effect of illumination duration was numerically weaker at 60° (*F* = 0.074, *p* = 0.989) than at 120° (*F* = 0.627, *p* = 0.654); however, neither effect was statistically significant.

Comparative analysis further revealed that the effect of illumination duration on sensitivity differences was spectrum-dependent and varied across *E*-vector orientations. Under violet light, illumination duration significantly amplified the LPL-dominant difference at the 150° *E*-vector orientation, corresponding to the negative-value range, with the greatest effect observed at 03:30. At 240°, the LDPL-dominant difference, corresponding to the positive-value range, reached its numerically highest value at 01:30, although the duration-dependent effect was not statistically significant. No significant duration-dependent effect was observed at the remaining *E*-vector orientations. Under orange light, the LDPL-dominant differences at 30° and 240° corresponded to the positive-value range. The LDPL-dominant difference at 30° reached its numerically greatest value at 05:30, whereas the duration-dependent effect at 240° reached statistical significance. By contrast, at 270°, illumination duration significantly amplified the LPL-dominant difference, with the greatest effect observed at 05:30. No significant duration-dependent effect was observed at the other *E*-vector orientations. Collectively, these results indicate that the difference in locust polarotactic aggregation sensitivity between LPL and LDPL is jointly governed by spectral properties, *E*-vector orientation, and illumination duration.

### 3.2. Polarotactic Aggregation Response Characteristics of Locusts to LPL and LDPL

**Figure 3:**
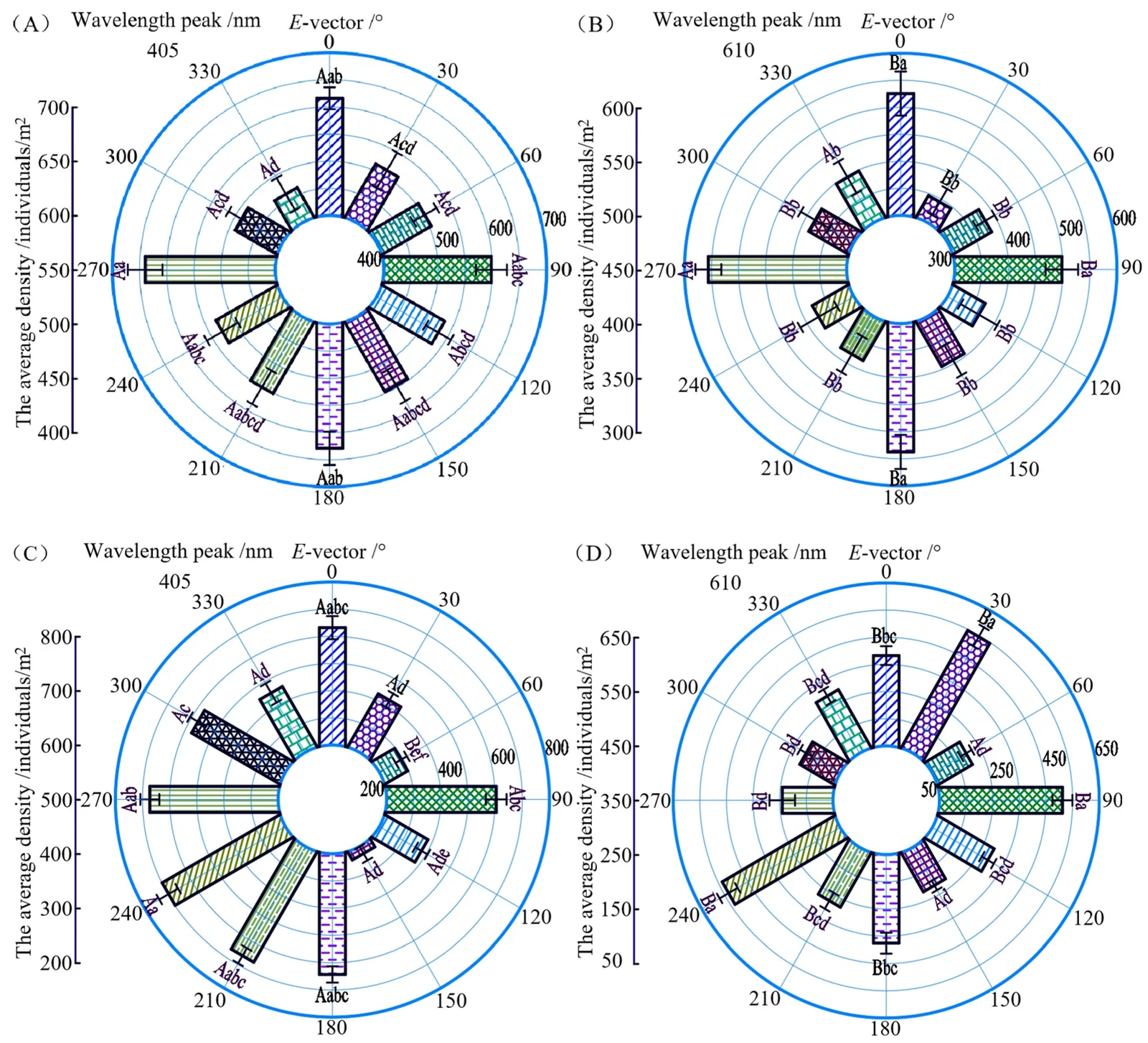
Vector response patterns of locust polarotactic aggregation to linearly polarized and linear detection polarized spectral light. (**A**) Linearly polarized violet light; (**B**) linearly polarized orange light; (**C**) linear detection-polarized violet light; (**D**) linear detection-polarized orange light. Note: The same lowercase letters indicate no significant difference in locust polarotactic aggregation among different *E*-vector orientations at the same peak wavelength (*p* > 0.05, Tukey’s HSD), whereas different lowercase letters indicate significant differences (*p* < 0.05, Tukey’s HSD). Between panels (**A**) and (**B**) (LPL), and between panels (**C**) and (**D**) (LDPL), the same uppercase letters indicate no significant difference in locust polarotactic aggregation between different peak wavelengths (405 nm vs. 610 nm) at the same *E*-vector orientation (*p* > 0.05, Tukey’s HSD), whereas different uppercase letters indicate significant differences (*p* < 0.05, Tukey’s HSD). Post hoc comparisons were conducted only after significant overall ANOVA results (*p* < 0.05).

**Figure 4:**
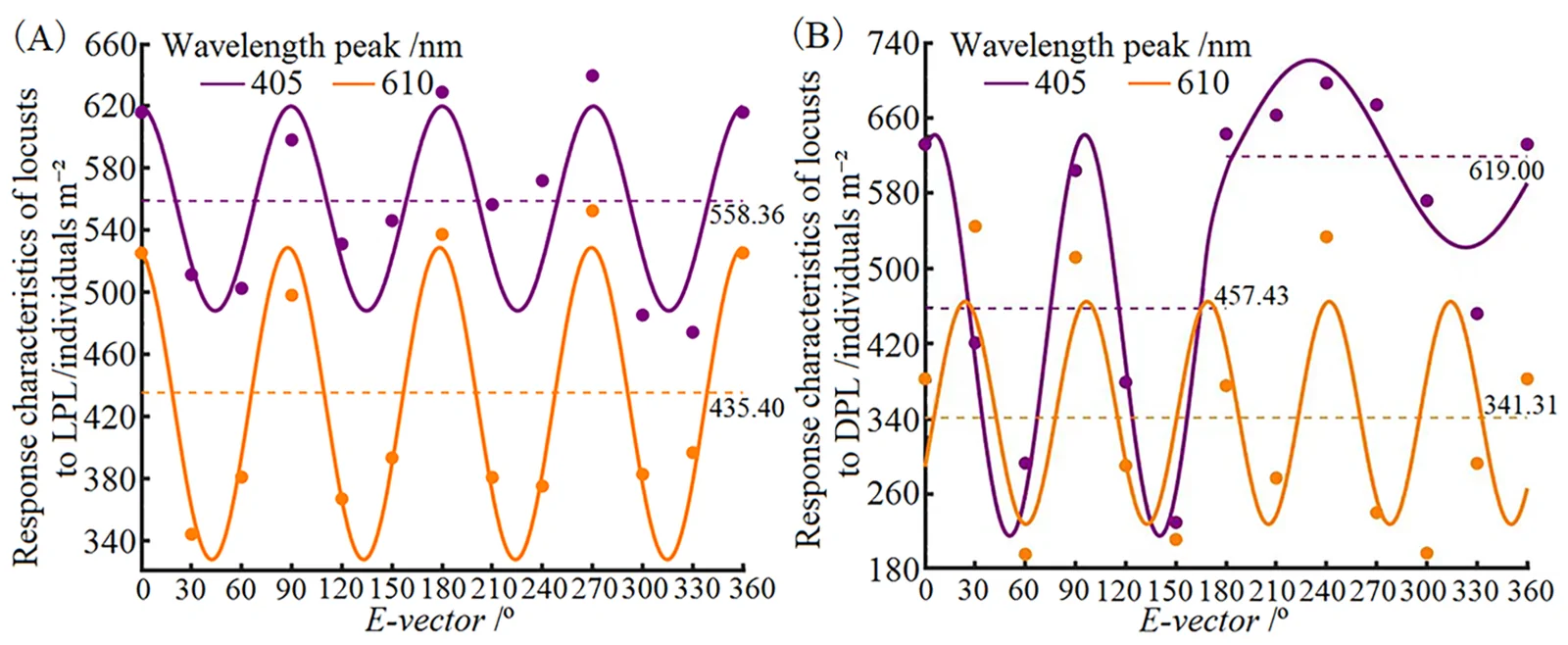
Response characteristics of locust polarotactic aggregation to linearly polarized and linear detection polarized spectral light. (**A**) Linearly polarized vector light; (**B**) linear detection-polarized vector light.

Under both LPL and LDPL illumination, the effect of *E*-vector orientation on locust polarotactic aggregation sensitivity was associated with the spectral properties of the polarized light stimulus (LPL: *p* < 0.001, *F* = 8.924 for violet light and *F* = 15.453 for orange light; LDPL: *p* < 0.001, *F* = 76.334 for violet light and *F* = 31.585 for orange light) (Figure 3). Under LPL, the spectral properties of linearly polarized light did not change the *E*-vector orientations associated with enhanced locust polarotactic aggregation sensitivity. *E*-vector orientations of 0°, 90°, 180° and 270° induced relatively strong responses, with the strongest response observed at 270°, followed by 180° (Figure 3A,B). Under LDPL, however, the spectral properties of linear detection-polarized light altered the *E*-vector orientations associated with enhanced locust polarotactic aggregation sensitivity. Under violet light, *E*-vector orientations of 0°, 90°, 180°, 240° and 270° induced relatively strong responses, with the strongest response observed at 240°, followed by 270°. Under orange light, *E*-vector orientations of 0°, 30°, 90°, 180° and 240° induced relatively strong responses, with the strongest response observed at 30°, followed by 240° (Figure 3C,D). In terms of response characteristics, locust sensitivity to *E*-vector orientation under LPL exhibited a stable cosine-periodic pattern that was independent of spectral properties. However, when violet and orange light were compared at the same *E*-vector orientation, aggregation sensitivity under violet light was higher than that under orange light (Figure 4A). Under LDPL, the polarotactic aggregation response showed spectrum-dependent sine/cosine tuning. Specifically, under violet light, the 0–180° *E*-vector range exhibited a two-cycle cosine-tuned response, whereas the 180–360° *E*-vector range exhibited a single-cycle sine-tuned response. Under orange light, the 0–360° *E*-vector range exhibited a five-cycle sine-tuned response (Figure 4B). At the 30° *E*-vector orientation, aggregation sensitivity was higher under orange light than under violet light, whereas at the remaining *E*-vector orientations, aggregation sensitivity was higher under violet light than under orange light.

LPL and LDPL differed markedly in their *E*-vector-dependent aggregation patterns and response characteristics, and the magnitude of these differences was associated with the spectral properties of the polarized light stimulus (Figure 3 and Figure 4). Under violet light, LDPL elicited stronger aggregation responses than LPL at *E*-vector orientations of 0°, 90°, 180° and 270°, although these differences were not statistically significant (*p* > 0.05). At 210°, 240° and 300°, LDPL induced significantly stronger responses than LPL (*p* < 0.05), whereas at 330°, LPL elicited a slightly stronger response than LDPL, although the difference was not significant (*p* > 0.05). At the remaining *E*-vector orientations, LPL induced significantly stronger responses than LDPL. Under orange light, LDPL elicited a stronger response than LPL at 90°, but the difference was not significant. At 30° and 240°, LDPL induced significantly stronger responses than LPL, whereas LPL induced significantly stronger responses at the remaining *E*-vector orientations. Cross-spectral comparisons further revealed no statistically significant differences between orange LDPL and violet LPL at either 30° or 240° (*p* > 0.05), although the numerical trends differed between the two angles. At the remaining E-vector orientations, violet LPL induced significantly stronger responses than orange LDPL (*p* < 0.05). When violet LDPL was compared with orange LPL, orange LPL induced a significantly stronger response at 30° (*p* < 0.05), whereas at 120°, the numerical difference favoring violet LDPL did not reach statistical significance (*p* > 0.05). At the remaining *E*-vector orientations, violet LDPL induced significantly stronger responses than orange LPL. Further comparison of the response characteristics induced by LPL and LDPL showed that the sine/cosine tuning properties and periodicity of locust polarotactic aggregation responses differed substantially under both matched and mismatched spectral conditions.

These results indicate that the polarotactic aggregation response of locusts, including both *E*-vector-dependent response patterns and tuning features, was shaped by the polarized light configuration, namely whether the stimulus was delivered as LPL or LDPL. In terms of response patterns, LPL did not alter the *E*-vector orientations associated with enhanced polarotactic aggregation sensitivity, whereas the spectral properties of LDPL markedly shifted the sensitivity-related *E*-vector orientations. In terms of response features, LPL maintained a stable cosine-periodic tuning pattern, whereas LDPL induced spectrum-dependent sine/cosine tuning. Overall, the spectral properties of the polarized light stimulus determined both the magnitude of locust polarotactic aggregation sensitivity and the associated sine/cosine tuning profile, including tuning form and response periodicity, with the regulatory effect being particularly pronounced under LDPL.

### 3.3. Light-Induced Polarotactic Aggregation Sensitivity of Locusts Under LPL and LDPL

Under both LPL and LDPL illumination, when the illumination duration was the same, the effect of *E*-vector orientation on locust polarotactic aggregation sensitivity was associated with the polarized spectral properties of the light stimulus (Table 3, Table 4, Table 5 and Table 6). Under linearly polarized violet light, the effect of E-vector orientation was significant at 03:30 (*F* = 5.632, *p* = 0.004; Table 3) but not at 23:30 (*F* = 2.116, *p* = 0.116; Table 3). Under linearly polarized orange light, significant effects were observed across all time points (*p* < 0.001; *F* = 15.706–24.643; Table 5). Under linear detection polarized violet light, significant effects were also observed across all time points (*p* < 0.005; *F* = 43.589–84.748; Table 4). Under linear detection polarized orange light, significant effects were observed across all time points (*p* < 0.001; *F* = 21.765–40.613; Table 6). Comparison between LPL and LDPL showed that, under the same spectral condition, the sensitivity difference varied more markedly under violet light; under different spectral conditions, the most pronounced sensitivity difference occurred under linear detection-polarized violet light.

In terms of light-induced aggregation sensitivity, LPL induced relatively high locust polarotactic aggregation density at *E*-vector orientations of 0°, 90°, 180°, and 270°, with no significant differences detected among these orientations. Under LDPL, relatively high polarotactic aggregation sensitivity was observed at *E*-vector orientations of 180°, 240°, and 270° under violet light, and at 30°, 90°, and 240° under orange light. Among these orientations, the numerical values were ranked as 270° > 180° > 90° ≈ 0° under LPL; as 240° > 270° > 180° under violet LDPL; and as 30° > 240° > 90° under orange LDPL. However, it should be noted that these numerical rankings do not necessarily imply statistically significant differences among the compared orientations, as detailed in Table 3, Table 4, Table 5 and Table 6. Moreover, under LPL, light-induced aggregation sensitivity under violet light was significantly higher than that under orange light at the same *E*-vector orientation (*p* < 0.05). Under LDPL, light-induced aggregation sensitivity under orange light was significantly higher than that under violet light at 30°, whereas violet light produced stronger light-induced aggregation sensitivity at the remaining *E*-vector orientations.

Under the same *E*-vector orientation, locust polarotactic aggregation sensitivity increased with illumination duration under both LPL and LDPL, reaching the highest level at 05:30. However, the magnitude of duration-dependent enhancement varied with the spectral properties of the polarized light stimulus. Under violet light, the enhancement effect of illumination duration was most pronounced at the 180° *E*-vector orientation under both LPL (*F* = 36.284, *p* < 0.001) and LDPL (*F* = 33.139, *p* < 0.001), whereas the increase from 03:30 to 05:30 was not significant. Under orange light, the duration-dependent enhancement was strongest at 180° under LPL (*F* = 23.186, *p* < 0.001) and at 240° under LDPL (*F* = 16.272, *p* < 0.001). Moreover, the increase from 03:30 to 05:30 was significant under orange light (*p* < 0.05).

Under the same illumination duration, the effect of spectral properties on locust polarotactic aggregation sensitivity was associated with both the polarized light configuration and the *E*-vector orientation. Under LPL, light-induced aggregation sensitivity was significantly higher under violet light than under orange light at all *E*-vector orientations (*p* < 0.05). Under LDPL, however, the spectral dependence of light-induced aggregation sensitivity varied with *E*-vector orientation. Specifically, at the 30° *E*-vector orientation, light-induced aggregation sensitivity was significantly higher under orange light than under violet light, whereas at the 150° *E*-vector orientation, no significant difference was observed between the two spectral conditions. At the remaining *E*-vector orientations, light-induced aggregation sensitivity was significantly higher under violet light than under orange light (*p* < 0.05).

When linearly polarized violet light was compared with linear detection polarized orange light, orange LDPL elicited significantly higher light-induced aggregation sensitivity at the 30° *E*-vector orientation, whereas violet LPL produced higher sensitivity at the remaining *E*-vector orientations. When linear detection polarized violet light was compared with linearly polarized orange light, orange LPL produced higher light-induced aggregation sensitivity at the 150° *E*-vector orientation, whereas violet LDPL produced higher sensitivity at the remaining *E*-vector orientations.

These results indicate that the spectral properties of LPL did not alter either the *E*-vector orientations associated with enhanced locust polarotactic aggregation sensitivity or their ranking, which consistently followed the order of 270°, 180°, 0°, and 90°. In contrast, the spectral properties of LDPL markedly changed both the composition and ranking of the sensitivity-related *E*-vector orientations, with the order of 240°, 270°, 180°, and 0° under violet light and 30°, 240°, and 90° under orange light. In addition, the duration-dependent regulatory enhancement was stronger under orange light, whereas the duration-dependent light-induced enhancement was more pronounced under violet light. Under orange light, LPL produced the numerically highest sensitivity at the 270° *E*-vector orientation, followed by 180°, whereas LDPL produced the numerically highest sensitivity at the 30° *E*-vector orientation, followed by 240°. Under violet light, LPL produced the numerically highest sensitivity at the 270° *E*-vector orientation, followed by 180°, whereas LDPL produced the numerically highest sensitivity at the 240° *E*-vector orientation, followed by 270°. Overall, comparisons across the two polarized light configurations, namely LPL and LDPL, and the two spectral conditions, namely violet and orange light, showed that, under the same illumination duration, violet LDPL at the 240° *E*-vector orientation produced the highest light-induced aggregation sensitivity, followed by violet LDPL at the 270° *E*-vector orientation.

## 4. Discussion

The tuning of locust central complex (CX) neurons to changes in the angle of polarization (AOP) within their receptive fields is matched to the skylight polarization pattern. By integrating unpolarized visual information with solar azimuth cues, *E*-vector orientation, wavelength information, polarization type, and polarization intensity, the locust visual system can enhance target contrast and generate robust polarization-based orientation. Accordingly, the comparative perception and subsequent integration of polarized light intensity, *E*-vector orientation, spectral gradients, and polarization patterns are critical for shaping polarization-mediated behavioral responses in locusts [23]. However, how polarized light configurations and spectral properties jointly regulate locust polarotactic aggregation sensitivity remains unclear, which limits the practical application of polarized light-based induction strategies for locust control. The present results showed that, under the same illumination duration, among the *E*-vector orientations at which LPL elicited higher sensitivity than LDPL, the largest light-induced difference occurred at 150° under violet light and at 270° under orange light. By contrast, among the *E*-vector orientations at which LDPL elicited higher sensitivity than LPL, the largest light-induced difference occurred at 240° under both violet and orange light. These findings are consistent with the ability of locust polarization vision to resolve heterogeneous polarized light information and with the polarization-dependent spectral guidance mechanism through which locusts selectively respond to specific *E*-vector orientations in changing polarization scenes [24].

Previous studies have shown that the dorsal rim area (DRA) of locusts and other insects mainly contains ultraviolet- and green-sensitive photoreceptors, whose polarization sensitivity differs markedly among wavelengths [25]. The violet light used in this study, with a peak wavelength of 405 nm, is close to the ultraviolet-sensitive range of the locust DRA and may therefore activate polarization-sensitive neurons more effectively. By contrast, orange light, with a peak wavelength of 610 nm, may act predominantly through non-DRA visual pathways and may involve cross-channel integration with green- or blue-sensitive photoreceptor inputs. These polarization-related signals are ultimately integrated in the central complex (CX) [26]. Therefore, the pronounced advantage of LDPL under violet light may be associated with high-gain encoding of ultraviolet-related polarized signals in the DRA, whereas the superior effect of LPL under orange light may be related to preferential CX responses to high-contrast polarization boundaries. In addition, LDPL introduces light intensity attenuation through dual-polarizer modulation according to Malus’ law, resulting in nonlinear intensity changes as a function of *E*-vector angle. This direction–intensity coupling may be encoded by the locust visual system as a simulated heterogeneous polarized light environment, thereby triggering a polarotactic behavioral pattern distinct from that induced by single-polarizer LPL.

In this study, LPL did not alter the cosine-periodic response of locusts to the *E*-vector, whereas LDPL induced spectrum-dependent trigonometric tuning. The shift in trigonometric form was most pronounced under violet light, whereas the change in response periodicity was most evident under orange light. This finding further supports the dominant role of spectral patterns in insect sensory processing during polarized-target detection [27] and extends the concept of polarization-opponent tuning in locusts. Previous studies have shown that polarization-sensitive neurons in locusts exhibit sinusoidal excitatory and inhibitory opponent responses within the 0–180° range [28,29]. In the present study, the observation range was extended to 0–360°, revealing that the trigonometric tuning induced by LDPL may result from the combined effects of *E*-vector orientation and Malus-law-governed intensity modulation. Together, these two factors form a direction–intensity-coupled stimulus. When polarized light input carries coupled information on both direction and intensity, locust CX neurons may process this imbalanced AOP input through an orthogonal decoding strategy [30,31], thereby shifting the response pattern from cosine to sine tuning. The pronounced shift in trigonometric form under violet light may be associated with the high polarization-sensitive gain of ultraviolet photoreceptors in the DRA, whereas the pronounced change in response periodicity under orange light may reflect modulation by green- or blue-sensitive channels in non-DRA regions during cross-spectral integration.

The present results indicate that the enhancement of locust polarotactic aggregation sensitivity by illumination duration was spectrally specific. The duration-dependent regulatory enhancement was more pronounced under orange light and peaked at 05:30, whereas violet light produced a stronger light-induced aggregation response. Specifically, among the *E*-vector conditions under which LPL elicited higher sensitivity than LDPL, the greatest enhancement occurred at 150° under violet light at 03:30 and at 270° under orange light at 05:30. Among the *E*-vector conditions under which LDPL elicited higher sensitivity than LPL, the greatest enhancement occurred at 240° under violet light at 01:30 and at 30° under orange light at 05:30. This temporal threshold effect is consistent with the time-compensated, azimuth-dependent response mechanisms of locust CX neurons [32,33]. Previous studies have shown that neurons in the locust central brain can integrate temporally accumulated polarized light information and perform directional compensation in response to time-dependent shifts in solar azimuth [34,35]. Therefore, the cumulative effect of nocturnal illumination may modulate the sensitivity threshold of the locust visual system. Shorter exposure durations, such as that corresponding to 01:30, may preferentially activate rapidly responding polarization-opponent channels, whereas prolonged exposure, such as that corresponding to 05:30, may progressively recruit additional CX neurons for signal integration, thereby generating stronger polarotactic outputs. The particularly pronounced regulatory enhancement observed under orange light at 05:30 may be associated with the crepuscular activity rhythm of locusts. Because orange light at 610 nm approximates a major spectral component of twilight skylight, prolonged exposure to this wavelength may induce greater visual adaptation and response gain in the locust visual system.

Compared with other insects, the long-term cumulative temporal feature observed in locust polarotactic aggregation appears to be a distinctive aspect of the polarization-mediated aggregation response in locusts. For example, polarization-based orientation in honeybees depends on an instantaneous *E*-vector detection strategy, allowing *E*-vector information to be assessed within seconds without prolonged light exposure [36]. In nocturnal dung beetles, polarization sensitivity is mainly governed by a degree-of-polarization threshold mechanism: their orientation performance is constrained by moon phase-dependent changes in the degree of polarization and becomes markedly impaired when the degree of polarization falls below 0.23, rather than being determined by illumination duration itself [37].

Unlike the instantaneous *E*-vector detection strategy of honeybees, which operates on a seconds-scale response window, and the degree-of-polarization threshold mechanism of nocturnal dung beetles [38,39], the present study showed that locust polarotactic aggregation sensitivity exhibited a distinct long-term cumulative temporal threshold feature, with key response time points at 01:30, 03:30, and 05:30. The peak response emerged only after several hours of accumulated illumination. This difference suggests that insects may adopt diverse temporal processing strategies for polarized light information in accordance with their ecological demands. Honeybees require rapid orientation during short daytime flights, dung beetles rely on moonlight polarization gradients for nocturnal navigation, whereas nocturnal aggregation in locusts may involve a polarization–intensity integration mechanism that depends on prolonged light exposure.

Based on these findings, this study provides clear parameter references for the intelligent design of polarized-spectrum induction light sources for locust control. Given the significant differences in the optimal sensitivity-associated *E*-vector orientations of locusts under LPL and LDPL across different spectral conditions, *E*-vector combinations can be optimized to maximize polarotactic induction efficiency. Specifically, under violet light, LDPL at the 240° *E*-vector orientation induced the strongest polarotactic aggregation sensitivity, followed by 270°; under orange light, LPL at the 270° *E*-vector orientation induced the strongest sensitivity, followed by LDPL at 30°. Therefore, combining these *E*-vector conditions may effectively enhance locust polarotactic responses. Because illumination duration is a key factor regulating sensitivity, differentiated optimal regulation strategies for violet and orange polarized light vector illumination can be established using 05:30, when sensitivity reached its maximum, as a critical temporal threshold. Based on the three temporal thresholds identified at 01:30, 03:30, and 05:30, a time-dependent switching strategy for polarized optical path configurations can be developed. During the early nocturnal phase from 19:30 to 01:30, the strategy should focus on violet LDPL at 240° and 30°. During the later nocturnal phase from 01:30 to 05:30, priority should be given to a combined configuration of orange LPL at 270° and orange LDPL at 30°. This temporally optimized configuration may enable precise and efficient polarized light induction output. Collectively, these results provide both a theoretical basis and a technical pathway for developing efficient and highly targeted polarized-spectrum trapping devices for locust control.

Although this study demonstrates that differences in locust polarotactic aggregation sensitivity between LPL and LDPL are jointly regulated by spectral properties, *E*-vector orientation, and illumination duration, several limitations should be considered when interpreting and applying these findings. This study identified sensitivity-associated *E*-vector orientations for locust polarotactic aggregation, including LPL orientations of 270°, 180°, 0°, and 90°; LDPL orientations of 240°, 270°, 180°, and 0° under violet light; and LDPL orientations of 30°, 240°, and 90° under orange light. We further propose that the trigonometric response induced by LDPL may arise from nonlinear orthogonal decoding of imbalanced AOP inputs by locust polarization-opponent neurons, and that temporal threshold effects, such as the response observed at 05:30, may be associated with time-compensated, azimuth-dependent CX neuronal responses and the accumulation of light-induced physiological states. These findings highlight the relevance of the present results for the design of intelligent polarized-spectrum light sources. First, the experiments were conducted in mesh-enclosed rearing sheds. Although nocturnal natural light was retained, this setting cannot fully reproduce the heterogeneous polarized light fields generated in natural habitats by vegetation reflection, water surface scattering, and other environmental optical processes; therefore, the potential interference of background polarized light in natural environments requires further evaluation [40,41]. Second, this study was restricted to healthy adult *L. migratoria manilensis* individuals within one week after emergence and did not examine individual variation associated with age, sex, or physiological state, such as starvation or mating status. Because polarization-vision sensitivity in locusts may vary with age or sex, caution is warranted when extending the present conclusions to other developmental stages or populations. Third, the mechanistic interpretation was based on behavioral measurements and inferences from existing literature, without direct electrophysiological recordings from the DRA or central complex (CX). Thus, the proposed neural encoding mechanisms underlying trigonometric tuning and temporal thresholds remain hypothetical and require further validation through electrophysiological experiments. Despite these limitations, this study systematically reveals the spectral dependence and temporal threshold characteristics of locust polarotactic aggregation sensitivity under the two polarized light configurations, LPL and LDPL. The identified sensitivity-associated *E*-vector orientations and illumination-duration-dependent regulatory patterns provide practical guidance for the engineering development of intelligent polarized light induction sources for locust control.

## 5. Conclusions

In this study, the polarotactic aggregation responses of *L. migratoria manilensis* were systematically investigated under vector-controlled violet and orange illumination delivered as either LPL or LDPL. Comparative analyses showed that illumination duration significantly enhanced locust polarotactic aggregation sensitivity and that this enhancement followed a distinct illumination-duration-threshold regulatory pattern. The spectral properties of LPL did not alter the *E*-vector-sensitive response pattern of locusts or their cosine-periodic tuning, whereas the spectral properties of LDPL markedly reshaped the *E*-vector-sensitive response pattern and induced spectrum-dependent trigonometric tuning. Specifically, violet light produced pronounced shifts in trigonometric form, whereas orange light led to substantial changes in *E*-vector-response periodicity. Nevertheless, the polarized light configuration, namely LPL or LDPL, did not alter the overall tendency for violet light to elicit higher sensitivity than orange light at sensitivity-associated *E*-vector orientations. This finding is important for defining the technical parameters and application modes of polarized light induction in locust control and provides behavioral evidence for elucidating the sensitivity mechanisms underlying locust polarotactic orientation and the neurobiological response characteristics of locust polarization vision. The aggregation results further confirmed that locusts can modulate their light-induced sensitivity to illumination duration according to the polarized light vector configuration and can use the visual sensitivity properties of polarized spectra to regulate the enhancement effect of illumination duration. Together, these findings provide behavioral evidence for the coordinated use of the polarization compass and azimuth compass during spatial orientation and navigation in locusts. It should be noted, however, that the present study was limited to behavioral observations, and the visual physiological mechanisms underlying locust polarotactic aggregation require further investigation.

## Figures and Tables

**Table 1:** Differences in locust polarotactic aggregation sensitivity between linear-detection polarized violet light (LDPL-Violet) and linearly polarized violet light (LPL-Violet).

Variable		Difference in Locust Polarotactic Aggregation Density/Individuals/m^2^					*F*	*p*
Time/(h)		21:30	23:30	1:30	3:30	5:30	*df* = 4	
Vector /(°)	0	25.00 ± 8.66 bcA	20.00 ± 12.15 bcA	40.00 ± 14.15 bcA	10.00 ± 5.92 cA	10.00 ± 4.38 bcA	0.264	0.895
	30	−105.00 ± 15.28 dB	−97.00 ± 13.32 deAB	−57.67 ± 13.07 cdA	−72.00 ± 11.37 dA	−90.33 ± 11.02 cA	1.670	0.232
	60	−181.00 ± 16.50 dA	−210.00 ± 15.52 efA	−226.00 ± 32.74 eA	−211.00 ± 20.82 eA	−220.00 ± 19.05 dA	0.404	0.802
	90	25.00 ± 8.43 bcA	20.00 ± 7.71 bcA	5.00 ± 3.65 bcA	−10.00 ± 5.82 cdA	−11.00 ± 5.12 cA	0.280	0.884
	120	−145.00 ± 18.44 dA	−155.00 ± 15.89 eA	−161.00 ± 18.08 deA	−155.00 ± 15.87 eA	−145.00 ± 22.30 dA	0.081	0.984
	150	−276.00 ± 20.72 eA	−298.00 ± 18.95 fA	−332.00 ± 32.00 eB	−340.00 ± 27.32 fB	−335.00 ± 28.66 eB	14.87	0.000
	180	20.00 ± 5.78 bcA	25.00 ± 7.85 bcA	25.00 ± 8.50 bcA	0.00 ± 2.89 cA	0.00 ± 3.00 bcA	0.657	0.636
	210	94.00 ± 14.58 abA	105.00 ± 15.00 abA	109.00 ± 18.52 abA	114.00 ± 17.51 aA	110.00 ± 18.87 aA	0.152	0.958
	240	110.00 ± 17.32 aA	125.00 ± 18.66 aA	140.00 ± 15.00 aA	125.00 ± 20.45 aA	91.67.00 ± 16.19 abA	1.553	0.260
	270	40.00 ± 12.43 abcA	42.00 ± 9.82 abcA	40.00 ± 10.88 bcA	25.00 ± 5.95 bcA	20.00 ± 5.78 abcA	1.449	0.288
	300	85.00 ± 15.00 abA	80.00 ± 15.00 abA	85.00 ± 18.87 abA	90.00 ± 11.55 abA	83.00 ± 13.36 abA	0.062	0.992
	330	−5.00 ± 2.89 cA	−5.00 ± 2.64 cdA	−25.00 ± 12.15 cA	−15.00 ± 7.94 cdA	−15.00 ± 9.63 cA	0.334	0.849
*F*	*df* = 11	49.319	39.895	30.134	101.583	35.682		
*p*		0.000	0.000	0.000	0.000	0.000		

Note: Positive values indicate a stronger induction effect of LDPL, whereas negative values indicate a stronger induction effect of LPL. Within the same column, the same lowercase letters indicate no significant difference in the difference in locust polarotactic aggregation density between LPL and LDPL under the same illumination time but different *E*-vector orientations (*p* > 0.05, Tukey’s HSD), whereas different lowercase letters indicate a significant difference (*p* < 0.05, Tukey’s HSD). Within the same row, the same uppercase letters indicate no significant difference in the difference in locust polarotactic aggregation density between LPL and LDPL under the same *E*-vector orientation but different illumination times (*p* > 0.05, Tukey’s HSD), whereas different uppercase letters indicate a significant difference (*p* < 0.05, Tukey’s HSD). The terms “strongest” and “followed by” in the main text refer to the numerical ranking of polarotactic aggregation density and do not necessarily indicate statistically significant differences.

**Table 2:** Difference in locust polarotactic aggregation density between linear detection polarized orange light (LDPL-Orange) and linearly polarized orange light (LPL-Orange).

Variable		Difference in Locust Polarotactic Aggregation Density/Individuals/m^2^					*F*	*p*
Time/h		21:30	23:30	1:30	3:30	5:30	*df* = 4	
Vector/(°)	0	134.00 ± 14.00 cdA	132.00 ± 12.06 cdA	141.00 ± 12.53 cdA	149.00 ± 17.32 dA	158.00 ± 16.36 deA	2.175	0.146
	30	−174.00 ± 19.12 aA	−186.00 ± 20.66 aAB	−199.00 ± 19.02 aA	−212.00 ± 18.85 aA	−233.00 ± 17.37 aA	1.013	0.446
	60	176.00 ± 17.01 cdA	184.00 ± 17.10 eA	189.00 ± 17.41 dA	188.00 ± 15.59 eA	187.00 ± 18.68 eA	0.074	0.989
	90	−1.00 ± 1.00 bA	−12.00 ± 5.65 bA	−19.00 ± 8.36 bA	−21.67 ± 8.21 bA	−14.00 ± 5.85 bA	0.657	0.635
	120	75.00 ± 12.41 bcA	73.00 ± 13.51 cA	74.00 ± 13.06 cA	84.00 ± 12.12 cA	77.00 ± 11.49 cA	0.627	0.654
	150	175.00 ± 15.31 cdA	177.00 ± 15.32 deA	188.00 ± 20.07 dA	188.00 ± 16.41 eA	180.00 ± 16.23 eA	0.133	0.967
	180	161.00 ± 16.03 cdA	151.00 ± 15.72 deA	161.00 ± 16.25 cdA	165.00 ± 17.35 deA	172.00 ± 16.93 eA	1.169	0.381
	210	89.00 ± 11.54 bcA	103.00 ± 10.60 cA	110.67 ± 14.05 cA	110.00 ± 11.53 cdA	102.00 ± 10.96 cA	0.788	0.559
	240	−135.67 ± 14.64 aA	−150.00 ± 16.06 aA	−189.33 ± 20.33 aA	−164.00 ± 16.25 bA	−183.00 ± 15.94 aA	3.493	0.049
	270	294.00 ± 17.12 eA	300.00 ± 21.06 fA	309.00 ± 23.00 eAB	323.00 ± 15.69 fAB	335.00 ± 21.73 fB	6.316	0.008
	300	186.00 ± 16.65 dA	191.00 ± 18.58 eA	189.00 ± 15.51 dA	174.00 ± 14.58 eA	187.00 ± 15.07 eA	0.753	0.579
	330	−136.33 ± 13.96 cdA	−99.00 ± 13.86 cA	−97.00 ± 10.54 cA	−105.00 ± 12.65 cA	−116.00 ± 11.87 cdA	0.726	0.594
*F*	*df* = 11	43.996	182.075	110.867	530.024	229.046		
*p*		0.000	0.000	0.000	0.000	0.000		

Note: Abbreviations and statistical details are as given in the notes to Table 1.

**Table 3:** Polarotactic aggregation sensitivity of locusts to linearly polarized violet light (LPL-Violet).

Variable		Locust Polarotactic Aggregation Density/Individuals/m^2^					*F*	*p*
Time/h		21:30	23:30	1:30	3:30	5:30	*df* = 4	
Vector/(°)	0	470.00 ± 40.00 aB	545.00 ± 43.00 aB	625.00 ± 43.30 aAB	710.00 ± 46.19 aA	730.00 ± 40.00 aA	10.602	0.001
	30	390 ± 22.91 aC	462.00 ± 36.39 aBC	531.00 ± 29.74 aAB	572.00 ± 31.43 bAB	602.00 ± 45.03 bA	10.861	0.001
	90	460.00 ± 26.46 aC	530.00 ± 39.26 aBC	605.00 ± 46.16 aAB	685.00 ± 42.72 abA	711.00 ± 39.55 abA	11.823	0.001
	150	416.00 ± 32.19 aC	488.00 ± 38.00 aB	572.00 ± 36.00 aAB	620.00 ± 26.46 abA	635.00 ± 35.00 abA	22.586	0.000
	180	480.00 ± 40.00 aD	560.00 ± 30.00 aCD	640.00 ± 26.46 aBC	725.00 ± 30.41 aAB	740.00 ± 30.00 aA	36.284	0.000
	240	450.00 ± 30.00 aD	510.00 ± 20.00b aCD	575.00 ± 40.00 aBC	655.00 ± 35.00 abAB	670.00 ± 40.00 abA	23.076	0.000
	270	495.00 ± 34.64 aB	568.00 ± 42.00 aB	650.00 ± 30.00 aA	735.00 ± 35.00 aA	750.00 ± 30.00 aA	21.203	0.000
*F*	*df* = 6	2.868	2.116	2.564	5.632	5.218		
*p*		0.049	0.116	0.069	0.004	0.005		

Note: Within the same column, identical lowercase letters indicate no significant difference in locust polarotactic aggregation density among different *E*-vector orientations at the same illumination time (*p* > 0.05, Tukey’s HSD), whereas different lowercase letters indicate a significant difference (*p* < 0.05, Tukey’s HSD). Within the same row, identical uppercase letters indicate no significant difference in locust polarotactic aggregation density among different illumination times under the same *E*-vector orientation (*p* > 0.05, Tukey’s HSD), whereas different uppercase letters indicate a significant difference (*p* < 0.05, Tukey’s HSD).

**Table 4:** Polarotactic aggregation sensitivity of locusts to linear detection polarized violet light (LDPL-Violet).

Variable		Locust Polarotactic Aggregation Density/Individuals/m^2^					*F*	*p*
Time/(h)		21:30	23:30	1:30	3:30	5:30	*df* = 4	
Vector/(°)	0	495.00 ± 25.00 aC	565.00 ± 30.00 aBC	640.00 ± 33.09 abAB	720.00 ± 43.14 aA	740.00 ± 42.72 aA	15.211	0.000
	30	285 ± 35.00 bC	365 ± 23.09 bBC	440 ± 30.00 cAB	500 ± 28.87 bA	515 ± 30.00 bA	19.511	0.000
	90	485 ± 41.15 aC	550 ± 20.00 aBC	610 ± 35.00 bAB	675 ± 25.98 aA	700 ± 37.32 aA	18.879	0.000
	150	140 ± 30.06 cC	190 ± 17.32 cBC	240 ± 25.00 dAB	280 ± 23.09 cA	300 ± 20.00 cA	14.576	0.000
	180	500 ± 30.48 aC	585 ± 31.45 aB	665 ± 37.32 abAB	725 ± 31.23 aA	740 ± 40.00 aA	33.139	0.000
	240	560 ± 27.32 aC	635 ± 35.65 aBC	715 ± 40.21 aAB	780 ± 40.00 aA	795 ± 31.75 aA	18.541	0.000
	270	535 ± 35.00 aC	610 ± 34.43 aBC	690 ± 30.34 abAB	760 ± 23.10 aA	770 ± 43.09 aA	24.070	0.000
*F*	*df* = 6	68.153	84.748	80.334	43.589	71.406		
*p*		0.000	0.000	0.000	0.000	0.000		

Note: Abbreviations and statistical details are as given in the notes to Table 3.

**Table 5:** Polarotactic aggregation sensitivity of locusts to linearly polarized orange light (LPL-Orange).

Variable		Locust Polarotactic Aggregation Density/Individuals/m^2^					*F*	*p*
Time/h		21:30	23:30	1:30	3:30	5:30	*df* = 4	
Vector/(°)	0	420.00 ± 33.18 aC	460.00 ± 34.14 aC	517.00 ± 33.51 aBC	580.00 ± 26.46 aAB	650.00 ± 30.95 aA	14.607	0.000
	30	261.00 ± 20.00 bC	299.00 ± 29.82 bC	341.00 ± 26.51 cBC	389.00 ± 36.35 bAB	431.00 ± 35.17 bA	15.261	0.000
	90	400.00 ± 30.00 aD	442.00 ± 19.08 aCD	490.00 ± 31.05 abBC	545.00 ± 30.12 aAB	614.00 ± 30.05 aA	14.221	0.000
	150	297.00 ± 23.90 bD	340.00 ± 35.38 bCD	396.00 ± 32.00 bcBC	445.00 ± 30.51 bAB	489.00 ± 38.97 bA	16.958	0.000
	180	435.00 ± 34.51 aD	475.00 ± 31.00 aC	528.00 ± 25.00 aBC	590.00 ± 30.27 aAB	659.00 ± 27.62 aA	23.186	0.000
	240	281.00 ± 30.51 bC	325.00 ± 29.00 bBC	375.00 ± 28.00 cAB	425.00 ± 39.51 bAB	470.00 ± 38.16 bA	10.918	0.001
	270	450.00 ± 28.51 aD	496.00 ± 32.58 aCD	546.00 ± 27.50 aBC	606.00 ± 35.38 aAB	665.00 ± 33.60 aA	17.984	0.000
*F*	*df* = 6	18.585	15.706	17.476	18.621	24.643		
*p*		0.000	0.000	0.000	0.000	0.000		

Note: Abbreviations and statistical details are as given in the notes to Table 3.

**Table 6:** Polarotactic aggregation sensitivity of locusts to linear detection polarized orange light (LDPL- Orange).

Variable		Locust Polarotactic Aggregation Density/Individuals/m^2^					*F*	*p*
Time/h		21:30	23:30	1:30	3:30	5:30	*df* = 4	
Vector/(°)	0	286.00 ± 25.58 bcC	328.00 ± 26.23 bBC	376.00 ± 39.40 bcAB	431.00 ± 26.29 bAB	492.00 ± 34.31 bA	10.401	0.001
	30	435.00 ± 34.24 aC	484.00 ± 38.31 aBC	540.00 ± 33.88 aABC	601.00 ± 37.51 aAB	664.00 ± 32.51 aA	7.050	0.001
	90	401.00 ± 22.11 abD	454.00 ± 40.33 aCD	509.00 ± 36.51 abBC	567.00 ± 32.00 aAB	628.00 ± 34.70 aA	16.276	0.000
	150	122.00 ± 24.06 cC	163.00 ± 37.00 cBC	208.00 ± 30.64 dABC	257.00 ± 35.79 cAB	309.00 ± 24.95 cA	9.075	0.001
	180	274.00 ± 25.48 bcC	324.00 ± 26.41 bC	367.00 ± 30.12 cdBC	425.00 ± 31.23 bAB	487.00 ± 33.51 bA	14.856	0.000
	240	420.00 ± 25.17 aD	475.00 ± 24.52 aCD	531.00 ± 32.93 aBC	589.00 ± 33.97 aAB	653.00 ± 38.51 aA	16.272	0.000
	270	156.00 ± 22.72 cB	196.00 ± 23.76 cB	237.00 ± 40.34 dAB	283.00 ± 24.27 cA	330.00 ± 38.16 cA	8.783	0.001
*F*	*df* = 6	21.765	33.152	23.828	35.168	40.613		
*p*		0.000	0.000	0.000	0.000	0.000		

Note: Abbreviations and statistical details are as given in the notes to Table 3.

## Data Availability

The original contributions presented in this study are included in the article. Further inquiries can be directed to the corresponding authors.

## Abbreviations

The following abbreviations are used in this manuscript:

LPL	Linearly polarized light
LDPL	Linear detection-polarized light
CX	Central complex
AOP	Angle of polarization
